# Supplementary material for: RAS-inhibiting biologics identify and probe druggable pockets including an SII-α3 allosteric site
Source: Nat Commun. 2021 Jun 30;12:4045. doi: 10.1038/s41467-021-24316-0 (PMC8245420; doi:10.1038/s41467-021-24316-0)
Supplement: Supplementary file 1 — Supplementary info [file 41467_2021_24316_MOESM1_ESM.pdf]

SUPPLEMENTARY DATA: RAS-inhibiting biologics identify and probe druggable pockets including an SII-α3 allosteric site.

SUPPLEMENTARY FIGURES

Supplementary Fig. 1. Affimer binding locations compared with SOS1 and RAF-RBD. a) Affimer K6 (green) occludes SOS1 (orange) binding RAS (grey). **b)** Affimer K6 (green) sterically clash with RAF-RBD (blue) binding of RAS (grey) **c)** Affimer K3 (magenta) occludes SOS1 (orange) binding RAS (grey). **d)** Affimer K3 (magenta) does not sterically clash with RAF-RBD (blue) binding of RAS (grey), but a conformational change to the Switch II region is seen compared to unbound KRASGDP. Images were generated in MacPyMOLv1.7.2.3 using RAS:SOS1 PDB:1BKD [http://doi.org/10.2210/pdb1BKD/pdb][1] and RAS:RAF-RBD PDB:4G0N [http://doi.org/10.2210/pdb4G0N/pdb][2].

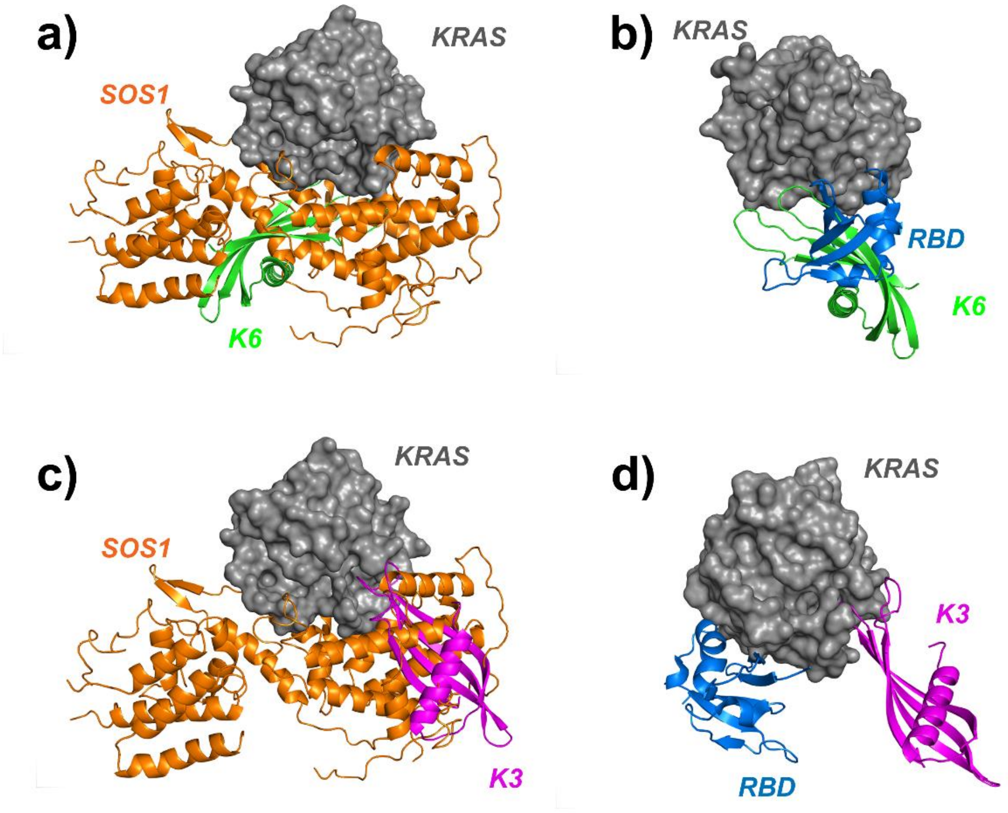

Supplementary Fig. 2. Affimer binding locations compared to other RAS binding biologics. Affimer K6 is shown in green, Affimer K3 in magenta and RAS in grey. **a)** Compared to NS1 monobody (red) that binds the α4-α5 interface. **b)** DARPins K13 and K19 (yellow and wheat respectively) that bind α3 and α4. **c)** DARPin K27 that spans across the SI/SII pocket (orange). **d)** Intrabody iDab6 (blue) that also spans across the SI/SII pocket**.** Images were generated in MacPyMOLv1.7.2.3 using PDB codes RAS:NS1-5E95 [http://doi.org/10.2210/pdb5E95/pdb][3]; RAS:DARPin K13-6H46 [http://doi.org/10.2210/pdb6H46/pdb][4]; RAS:DARPin K19-6H47 [http://doi.org/10.2210/pdb6H47/pdb][4]; RAS:DARPin K27-5O2S [http://doi.org/10.2210/pdb5O2S/pdb][5] and RAS:iDab6-2UZI [http://doi.org/10.2210/pdb2UZI/pdb][6]**.**

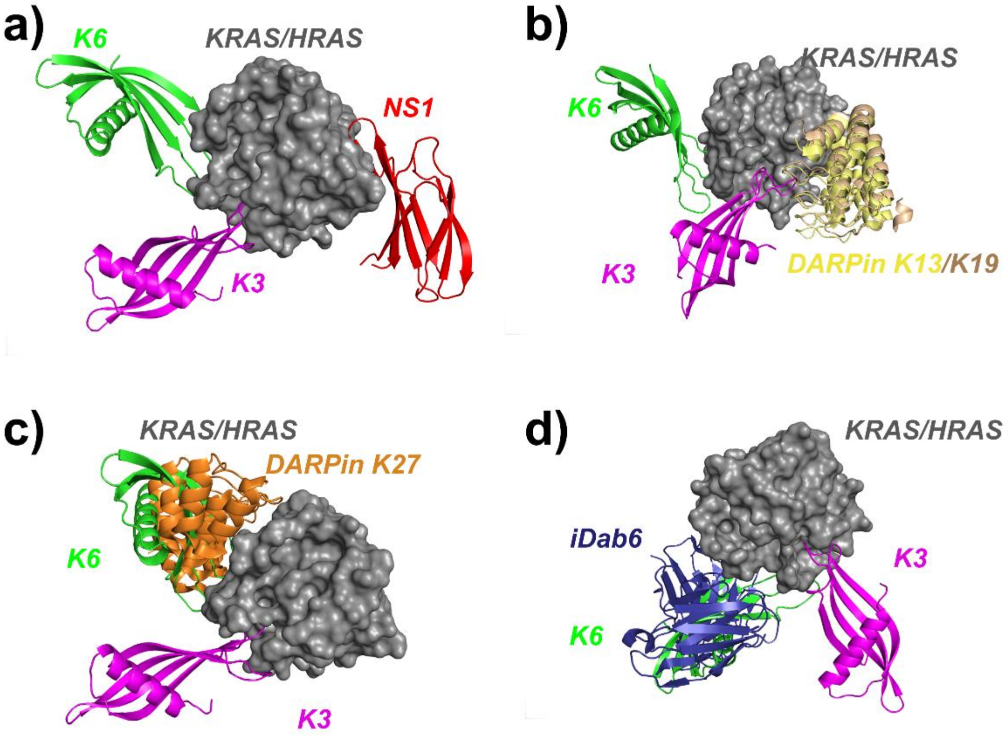

Supplementary Fig. 3. Uncropped images of all membranes shown in this paper.

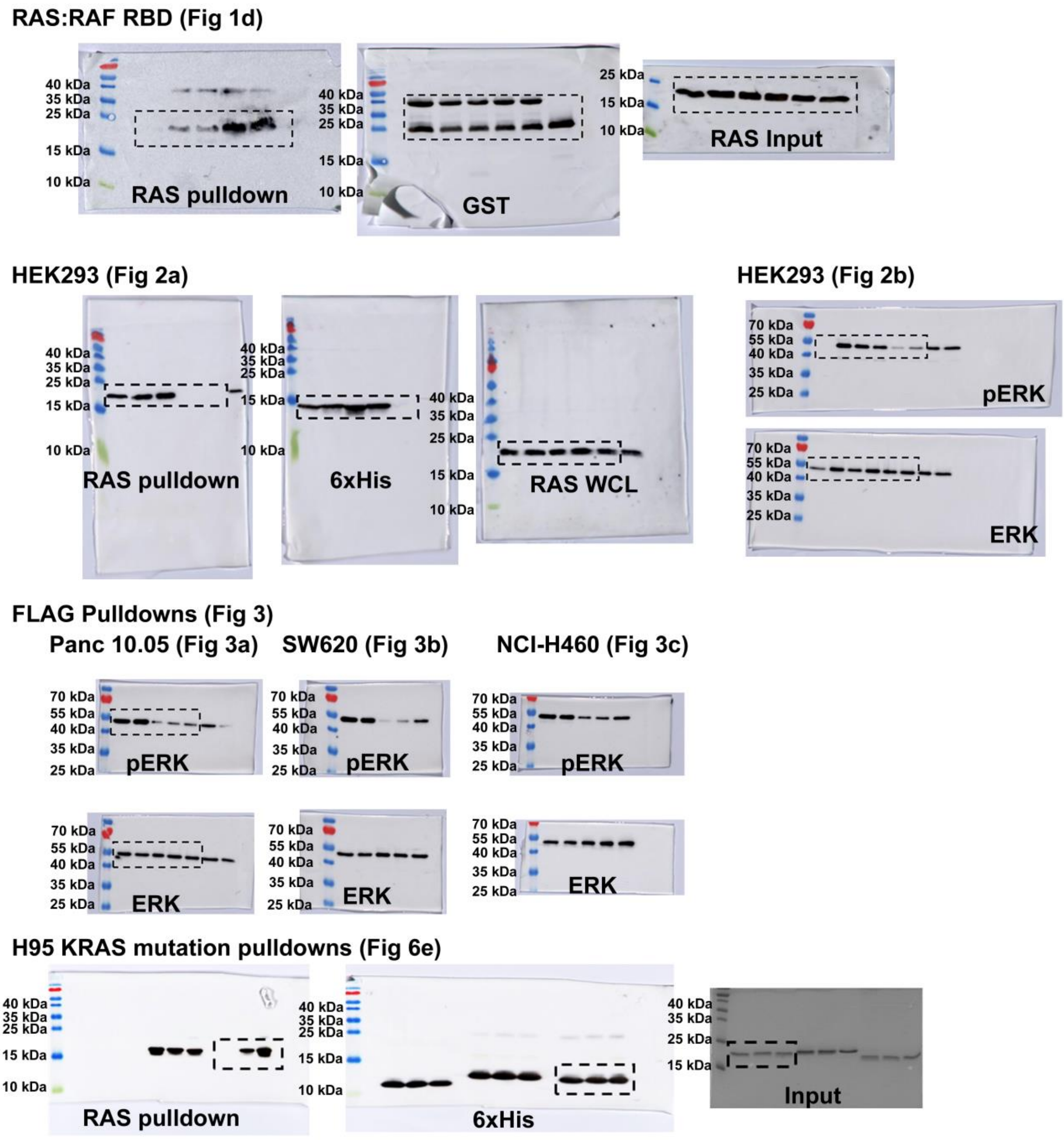

Supplementary Fig. 4. KRAS:Affimer complex formation. Size-exclusion chromatographs of KRAS:Affimer complexes, KRAS:K3 (a), KRAS:K6 (c) and KRAS:K69 (e). SDS-PAGE of fractions from size-exclusion peaks that were used for X-ray crystallography, KRAS:K3 (b), KRAS:K6 (d) (Every other fractions was run for KRAS:K6 complex) and KRAS:K69 (f) (Fractions 80-87 were not used for X-ray crystallography). (n = 1 independent experiment as complexes were taken forward for crystallography).

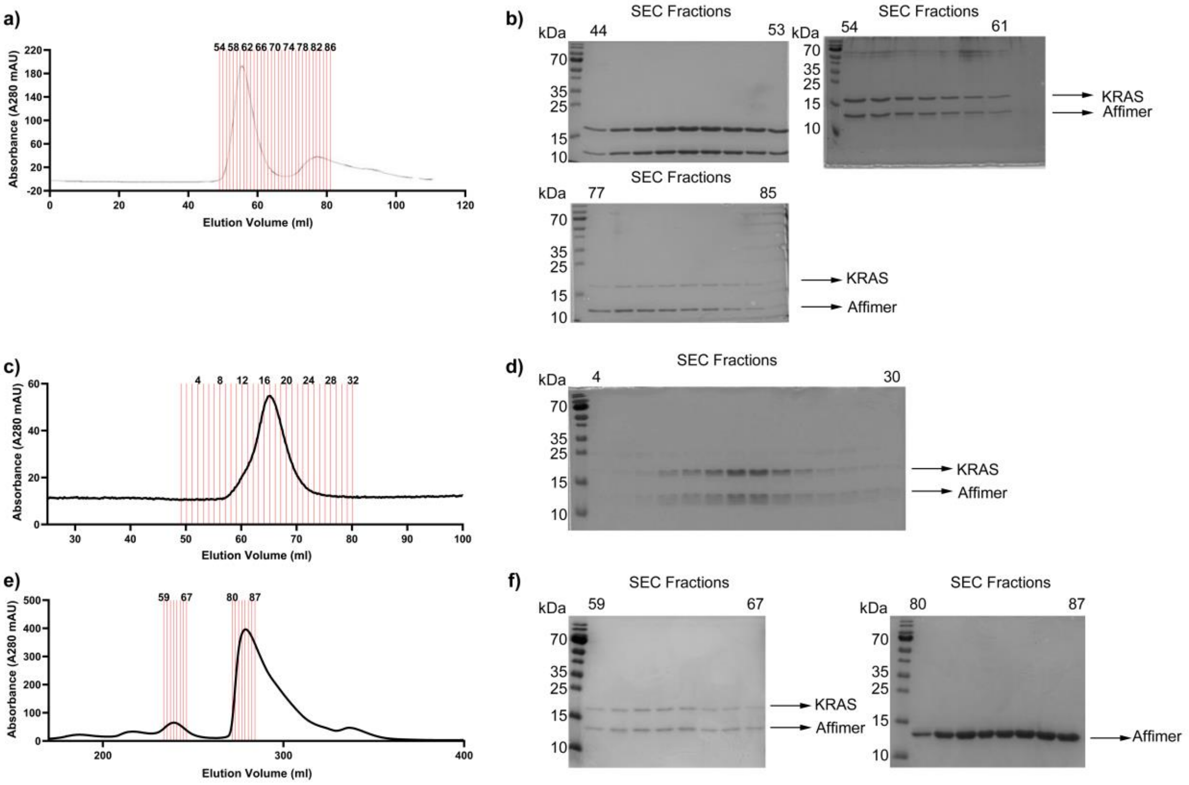

SUPPLEMENTARY TABLES

Supplementary Table 1. Amino acid sequences of variable regions of RAS-binding Affimers

Affimer	Variable Region 1	Variable Region 2
K3	HSIDIWYDF	KLNNSHTYK
K6	HFTPWFQRN	RIMVTDKMR
K19	FFYLWLAPG	AANSPMYHE
K37	QYNPWFQTN	VIHGTRWGN
K68	VYNPWYQVN	NMRVDMIVH
K69	WHFDYQQYN	RQLRMGSMN
K91	WDFSAWWKY	RNRYFKFPN

Supplementary Table 2. Affimer inhibition of SOS1-mediated nucleotide exchange. IC_{50} values calculated from 3-5 independent experiments. Data is mean ± SEM. Source data are provided as a Source Data file.

Affimer	IC_{50} values (nM)					
	KRAS wt	KRAS G12D	KRAS G12V	KRAS Q61H	HRAS wt	NRAS
K3	144 ± 94	144 ± 40	176 ± 115	3005 ± 865	2585 ± 335	No inhibition
K6	594 ± 271	185 ± 46	571 ± 148	532 ± 165	389 ± 187	477 ± 44
K37	697 ± 158	356 ± 161	640 ± 253	1075 ± 651	626 ± 320	647 ± 219

Supplementary Table 3. Binding Affinities and IC50 data for RAS binding compounds discussed in this paper.

	Binding Affinity (nM)	Method	IC_{50} (nM)	Assay	Reference
SI/SII-Pocket					
**Affimer K6**	1.36 ± 0.87 WT-KRAS^{GDP}<br>7.88 ± 1.09 WT-KRAS^{GppNHp}	SPR	592 ± 271	Nucleotide exchange	This paper
**Abd-7**	51	NMR	8×10^3– 1×10^4 in cells	Cell viability/<br>pERK assays	[1]
**BI-2852**	750	ITC	490	Nucleotide exchange	[2]
**DCAI**	1.1×10^6	NMR	1.55×10^3 ± 36	Nucleotide exchange	[3]
**Compound 13**	3.4×10^5	NMR	58 ±8 % reduction with 1mM compound	Nucleotide exchange	[10]
SII-Pocket					
**K3**	59.4 ± 15 WT-KRAS^{GDP}<br>44.4 ± 0.8 WT-KRAS^{GppNHp}	SPR	150	Nucleotide exchange	This paper
**KRpep-2d**	8.9 G12D-KRAS^{GDP}<br>11 G12D-KRAS^{GTP}<br>58 WT-KRAS^{GDP}<br>200 WT-KRAS^{GTP}	SPR	1.6 (with DTT G12D-KRAS)<br>87 (without DTT G12D-KRAS)<br>42 (with DTT WT-KRAS)	Nucleotide exchange	[5,6]
**AMG510**	Not measured (covalent binding)	N/A	90	Nucleotide exchange	[13]
**ARS1620**	Not measured (covalent binding)	N/A	900	Nucleotide exchange	[13]

Supplementary Table 4. X-ray crystallographic data collection and refinement statistics for Affimer-KRAS complexes. Values given in parentheses correspond to those in the outermost shell of the resolution range.

	KRAS-K3 (6YXW)	KRAS-K6 (6YR8)	KRAS-K69 (7NY8)
**Data collection**			
Space group	$P\ 2_1$	$I\ 4_1$	$P\ 2_1$
Cell dimensions			
a, *b*, *c* (Å)	73.1, 39.5, 113.1	71.6, 71.6, 144.4	39.01, 168.78, 39.65
α, β, γ (°)	90.0, 106.9, 90.0	90.0, 90.0, 90.0	90.0, 92.7, 90.0
Resolution (Å)	54.16 (2.06)	64.12 (1.90)	84.53 (1.80)
R_{sym} or R_{merge}	8.9 (39.9)	8.7 (269.0)	7.2 (73.4)
$I / \sigma I$	6.2 (1.3)	13.3 (0.6)	19.3 (1.7)
Completeness (%)	99.9	100.0	100.0
Redundancy	2.9 (3.0)	14.4 (11.4)	6.9 (6.9)
**Refinement**			
Resolution (Å)	54.16-2.06	64.12-1.90	84.53-1.80
No. of observed reflections	113731 (8427)	379142 (24154)	324059 (16141)
No. of unique reflections	38740 (2830)	28572 (2116)	47236 (2336)
R_{work} / R_{free}	0.236/0.278	0.211/0.246	0.154/0.210
No. atoms			
Protein	4137	2012	4569
Ligand/ion	58	29	58
Water	103	124	306
B-factors			
Protein	46.6	67.3	32.18
Ligand/ion	37.5	71.4	28.8
Water	45.4	68.2	36.1
R.m.s. deviations			
Bond lengths (Å)	0.0055	0.0086	0.0050
Bond angles (°)	1.0202	1.2904	1.3140

Supplementary Table 5. Primers used in this study

Primer name	Primer sequence 5' – 3'
KRAS – forward	CGCGCTAGCATGACCGAATATAAACTGGTGG
KRAS – reverse	CGTTGGCGGCCGCTTATTTATGTTTGCGAATTTCACG
Affimer-His-forward	ATGGATCCGCCACCATGGCCGCTACCGGTGTTCGTG
Affimer-His-reverse	GTTTGGCCAACCACTGCGACTAATATTTCTACTGCTACTGTTCGTAGTAGTAGT AGTAGTAATCCCATTCGCCGGCGATTACG
Affimer-GFP-forward	TATATGCGATCGCCATGGGTAACGAAAACTCCCTG
Affimer GFP-reverse	AATACGCGTAGCGTCACCAACCGGTTTG
K3-VR1.1 forward	GTTGTTAAAGCGAAAGAACAGGCTTCTATCGACATCTGGTACGAC
K3-VR1.1 reverse	GTCGTACCAGATGTCGATAGAAGCCTGTTCTTTCGCTTTAACAAC
K3-VR1.2 forward	AAAGCGAAAGAACAGCATGCTATCGACATCTGGTACG
K3-VR1.2 reverse	CGTACCAGATGTCGATAGCATGCTGTTCTTTCGCTTT
K3-VR1.3 forward	GTTAAAGCGAAAGAACAGCATTCTGCTGACATCTGGTACGACTTCACCATG
K3-VR1.3 reverse	CATGGTGAAGTCGTACCAGATGTCAGCAGAATGCTGTTCTTTCGCTTTAAC
K3-VR1.4 forward	CGAAAGAACAGCATTCTATCGCTATCTGGTACGACTTCACCAT
K3-VR1.4 reverse	ATGGTGAAGTCGTACCAGATAGCGATAGAATGCTGTTCTTTCG
K3-VR1.5 forward	CGAAAGAACAGCATTCTATCGACGCTTGGTACGACTTCACCATGTACTA
K3-VR1.5 reverse	TAGTACATGGTGAAGTCGTACCAAGCGTCGATAGAATGCTGTTCTTTCG
K3-VR1.6 forward	AGAACAGCATTCTATCGACATCGCTTACGACTTCACCATGTACTACC
K3-VR1.6 reverse	GGTAGTACATGGTGAAGTCGTAAGCGATGTCGATAGAATGCTGTTCT
K3-VR1.7 forward	CAGCATTCTATCGACATCTGGGCTGACTTCACCATGTACTACCTG
K3-VR1.7 reverse	CAGGTAGTACATGGTGAAGTCAGCCCAGATGTCGATAGAATGCTG
K3-VR1.8 forward	ATTCTATCGACATCTGGTACGCTTTCACCATGTACTACCTGAC

K3-VR1.8 reverse	GTCAGGTAGTACATGGTGAAAGCGTACCAGATGTCGATAGAAT
K3-VR1.9 forward	TCTATCGACATCTGGTACGACGCTACCATGTACTACCTGACCCTG
K3-VR1.9 reverse	CAGGGTCAGGTAGTACATGGTAGCGTCGTACCAGATGTCGATAGA
K3-VR2.1 forward	CTGTACGAAGCGAAAGTTTGGGTTAAGGCTCTGAACAACAGTCATACCTATAAAAAC
K3-VR2.1 reverse	GTTTTTATAGGTATGACTGTTGTTCAGAGCCTTAACCCAAACTTTCGCTTCGTACAG
K3-VR2.2 forward	GTACGAAGCGAAAGTTTGGGTTAAGAAAGCTAACAACAGTCATACCTATAAAAACTTC
K3-VR2.2 reverse	GAAGTTTTTATAGGTATGACTGTTGTTAGCTTTCTTAACCCAAACTTTCGCTTCGTAC
K3-VR2.3 forward	CGAAGCGAAAGTTTGGGTTAAGAAACTGGCTAACAGTCATACCTATAAAAACTTCAAAG
K3-VR2.3 reverse	CTTTGAAGTTTTTATAGGTATGACTGTTAGCCAGTTTCTTAACCCAAACTTTCGCTTCG
K3-VR2.4 forward	AGCGAAAGTTTGGGTTAAGAAACTGAACGCTAGTCATACCTATAAAAACTTCAAAGAAC
K3-VR2.4 reverse	GTTCTTTGAAGTTTTTATAGGTATGACTAGCGTTCAGTTTCTTAACCCAAACTTTCGCT
K3-VR2.5 forward	AGTTTGGGTTAAGAAACTGAACAACGCTCATACCTATAAAAACTTCAAAGAAC
K3-VR2.5 reverse	GTTCTTTGAAGTTTTTATAGGTATGAGCGTTGTTCAGTTTCTTAACCCAAACT
K3-VR2.6 forward	CGAAAGTTTGGGTTAAGAAACTGAACAACAGTGCTACCTATAAAAACTTCAAAG
K3-VR2.6 reverse	CTTTGAAGTTTTTATAGGTAGCACTGTTGTTCAGTTTCTTAACCCAAACTTTCG
K3-VR2.7 forward	GGTTAAGAAACTGAACAACAGTCATGCTTATAAAAACTTCAAAGAACTGCAGG
K3-VR2.7 reverse	CCTGCAGTTCTTTGAAGTTTTTATAAGCATGACTGTTGTTCAGTTTCTTAACC
K3-VR2.8 forward	AAGAAACTGAACAACAGTCATACCGCTAAAAACTTCAAAGAACTGCAGGAG
K3-VR2.8 reverse	CTCCTGCAGTTCTTTGAAGTTTTTAGCGGTATGACTGTTGTTCAGTTTCTT
K3-VR2.9 forward	AAGAAACTGAACAACAGTCATACCTATGCTAACTTCAAAGAACTGCAGGAGTTCAA
K3-VR2.9 reverse	TTGAACTCCTGCAGTTCTTTGAAGTTAGCATAGGTATGACTGTTGTTCAGTTTCTT

K6-VR1.1 forward	TGTTAAAGCGAAAGAACAGGCTTTCACTCCGTGGTTCCAG
K6-VR1.1 reverse	CTGGAACCACGGAGTGAAAGCCTGTTCTTTCGCTTTAACA
K6-VR1.2 forward	TCGTGTTGTTAAAGCGAAAGAACAGCATGCTATCCGTGGTTCCAG
K6-VR1.2 reverse	CTGGAACCACGGAGTAGCATGCTGTTCTTTCGCTTTAACAACACGA
K6-VR1.3 forward	GAAAGAACAGCATTTCGCTCCGTGGTTCCAGCG
K6-VR1.3 reverse	CGCTGGAACCACGGAGCGAAATGCTGTTCTTTC
K6-VR1.4 forward	TAAAGCGAAAGAACAGCATTTCACTGCTTGGTTCCAGCGTA
K6-VR1.4 reverse	TACGCTGGAACCAAGCAGTGAAATGCTGTTCTTTCGCTTTA
K6-VR1.5 forward	AAAGAACAGCATTTCACTCCGGCTTTCCAGCGTAACACCATGTAC
K6-VR1.5 reverse	GTACATGGTGTTACGCTGGAAAGCCGGAGTGAAATGCTGTTCTTT
K6-VR1.6 forward	GAACAGCATTTCACTCCGTGGGCTCAGCGTAACACCATGTACTAC
K6-VR1.6 reverse	GTAGTACATGGTGTTACGCTGAGCCCACGGAGTGAAATGCTGTTC
K6-VR1.7 forward	CAGCATTTCACTCCGTGGTTCGCTCGTAACACCATGTACTACCTG
K6-VR1.7 reverse	CAGGTAGTACATGGTGTTACGAGCGAACCACGGAGTGAAATGCTG
K6-VR1.8 forward	CACTCCGTGGTTCCAGGCTAACACCATGTACTACC
K6-VR1.8reverse	GGTAGTACATGGTGTTAGCCTGGAACCACGGAGTG
K6-VR1.9 forward	CATTTCACTCCGTGGTTCCAGCGTGCTACCATGTACTACCTGACC
K6-VR1.9 reverse	GGTCAGGTAGTACATGGTAGCACGCTGGAACCACGGAGTGAAATG
K6-VR2.1 forward	CTGTACGAAGCGAAAGTTTGGGTTAAGGCTATTATGGTTACCGATAAAATGAGA<br>AAC
K6-VR2.1 reverse	GTTTCTCATTTTATCGGTAACCATAATAGCCTTAACCCAAACTTTCGCTTCGTAC<br>AG
K6-VR2.2 forward	GAAGCGAAAGTTTGGGTTAAGTGTGCTATGGTTACCGATAAAATGAGAAAC
K6-VR2.2 reverse	GTTTCTCATTTTATCGGTAACCATAGCTCTCTTAACCCAAACTTTCGCTTC

K6-VR2.3 forward	TGTACGAAGCGAAAGTTTGGGTTAAGAGAATTGCTGTTACCGATAAAATGAGAAACT
K6-VR2.3 reverse	AGTTTCTCATTTTATCGGTAACAGACCTTCTCTTAACCCAAACTTTCGCTTCGTACA
K6-VR2.4 forward	AAAGTTTGGGTTAAGTGTTAATAGGCTACCGATAAAATGAGAAACTTC
K6-VR2.4 reverse	GAAGTTTCTCATTTTATCGGTAGCCATAATTCTCTTAACCCAAACTTT
K6-VR2.5 forward	AAAGTTTGGGTTAAGAGAATTATGGTTGCTGATAAAATGAGAAACTTCAAAGAACTG
K6-VR2.5 reverse	CAGTTCTTTGAAGTTTCTCATTTTATCAGCAACCATAATTCTCTTAACCCAAACTTT
K6-VR2.6 forward	GGTTAAGAGAATTATGGTTACCGCTAAAATGAGAAACTTAAAGAAC
K6-VR2.6 reverse	GTTCTTTGAAGTTTCTCATTTTAGCGGTAACCATAATTCTCTTAACC
K6-VR2.7 forward	GGGTTAAGAGAATTATGGTTACCGATGCTATGAGAAACTTCAAAGAACTGCAGG
K6-VR2.7 reverse	CCTGCAGTTCTTTGAAGTTTCTCATAGCATCGGTAACCATAATTCTCTTAACCC
K6-VR2.8 forward	GAAAGTTTGGGTTAAGTGAATTATGGTTACCGATAAAGCTAGAAACTTCAAAGAACTGC
K6-VR2.8reverse	GCAGTTCTTTGAAGTTTCTAGCTTTATCGGTAACCATAATTCTCTTAACCCAAACTTTC
K6-VR2.9 forward	AAGAGAATTATGGTTACCGATAAAATGGCTAACTTCAAAGAACTGCAGGAGTTCAAA
K6-VR2.9 reverse	TTTGAACTCCTGCAGTTATTTGAAGTTAGCCATTTTATCGGTAACCATAATTCTCTT
Affimer-VR1 forward	ATGGCTAGCAACTCCCTGGAAATCGAAG
Affimer-VR1 reverse	CACCGTCTTTAGCTTCCAGG
Affimer-VR2 forward	CCTGGAAGCTAAAGACGGTG
Affimer-VR2 reverse	TACCCTAGTGGTGATGATGGTGATGC

SUPPLEMENTARY REFERENCES

1. Quevedo, C.E. et al. Small molecule inhibitors of RAS-effector protein interactions derived using an intracellular antibody fragment. *Nat Commun* **9**, 3169 (2018).
2. Kessler, D. et al. Drugging an undruggable pocket on KRAS. *Proceedings of the National Academy of Sciences* **116**, 15823 (2019).
3. Maurer, T. et al. Small-molecule ligands bind to a distinct pocket in Ras and inhibit SOS-mediated nucleotide exchange activity. *Proc Natl Acad Sci U S A* **109**, 5299-304 (2012).
4. Sun, Q. et al. Discovery of small molecules that bind to K-Ras and inhibit Sos-mediated activation. *Angew Chem Int Ed Engl* **51**, 6140-3 (2012).
5. Niida, A. et al. Investigation of the structural requirements of K-Ras(G12D) selective inhibitory peptide KRpep-2d using alanine scans and cysteine bridging. *Bioorganic & Medicinal Chemistry Letters* **27**, 2757-2761 (2017).
6. Sogabe, S. et al. Crystal Structure of a Human K-Ras G12D Mutant in Complex with GDP and the Cyclic Inhibitory Peptide KRpep-2d. *ACS medicinal chemistry letters* **8**, 732-736 (2017).
7. Canon, J. et al. The clinical KRAS(G12C) inhibitor AMG 510 drives anti-tumour immunity. *Nature* **575**, 217-223 (2019).
8. Chen, V.B. et al. MolProbity: all-atom structure validation for macromolecular crystallography. *Acta crystallographica. Section D, Biological crystallography* **66**, 12-21 (2010).
