# Supplementary material for: RAS-inhibiting biologics identify and probe druggable pockets including an SII-α3 allosteric site
Source: Nat Commun. 2021 Jun 30;12:4045. doi: 10.1038/s41467-021-24316-0 (PMC8245420; doi:10.1038/s41467-021-24316-0)
Supplement: Supplementary file 3 — Reporting Summary [file 41467_2021_24316_MOESM3_ESM.pdf]

Corresponding author(s): Darren C Tomlinson
Last updated by author(s): May 20, 2021

Reporting Summary

Nature Research wishes to improve the reproducibility of the work that we publish. This form provides structure for consistency and transparency in reporting. For further information on Nature Research policies, see our Editorial Policies and the Editorial Policy Checklist.

Statistics

For all statistical analyses, confirm that the following items are present in the figure legend, table legend, main text, or Methods section.

n/a	Confirmed	
☐	☒	The exact sample size (*n*) for each experimental group/condition, given as a discrete number and unit of measurement
☐	☒	A statement on whether measurements were taken from distinct samples or whether the same sample was measured repeatedly
☐	☒	The statistical test(s) used AND whether they are one- or two-sided<br>*Only common tests should be described solely by name; describe more complex techniques in the Methods section.*
☒	☐	A description of all covariates tested
☐	☒	A description of any assumptions or corrections, such as tests of normality and adjustment for multiple comparisons
☐	☒	A full description of the statistical parameters including central tendency (e.g. means) or other basic estimates (e.g. regression coefficient) AND variation (e.g. standard deviation) or associated estimates of uncertainty (e.g. confidence intervals)
☐	☒	For null hypothesis testing, the test statistic (e.g. *F*, *t*, *r*) with confidence intervals, effect sizes, degrees of freedom and *P* value noted<br>*Give P values as exact values whenever suitable.*
☒	☐	For Bayesian analysis, information on the choice of priors and Markov chain Monte Carlo settings
☒	☐	For hierarchical and complex designs, identification of the appropriate level for tests and full reporting of outcomes
☒	☐	Estimates of effect sizes (e.g. Cohen's *d*, Pearson's *r*), indicating how they were calculated

Our web collection on statistics for biologists contains articles on many of the points above.

Software and code

Policy information about availability of computer code

Data collection	Data were collected with the following commercially available software: Tecan SPARKCONTROL v1.1Spark, CellReporterXpress v2.6, Harmony v4.6, BIAcore v2.9.
Data analysis	Data were analysed with the following commercial softwares: Origin Pro v9.7.0 , Graphpad Prism v9.1.0, ImageJ v1.52, BIAevaluation v2.9, Columbus v2.7.1, MetaExpress v6.7.

For manuscripts utilizing custom algorithms or software that are central to the research but not yet described in published literature, software must be made available to editors and reviewers. We strongly encourage code deposition in a community repository (e.g. GitHub). See the Nature Research guidelines for submitting code & software for further information.

Data

Policy information about availability of data

All manuscripts must include a data availability statement. This statement should provide the following information, where applicable:

- Accession codes, unique identifiers, or web links for publicly available datasets
- A list of figures that have associated raw data
- A description of any restrictions on data availability

The X-Ray crystal structures generated during and analysed during the current study are available in the PDB repository [https://www.rcsb.org] with the following codes: 6YXW [http://doi.org/10.2210/pdb6yxw/pdb], 6YR8 [http://doi.org/10.2210/pdb6YR8/pdb] and 7NY8 [https://doi.org/10.2210/pdb7NY8/pdb]. Source data are provided with this paper.

Field-specific reporting

Please select the one below that is the best fit for your research. If you are not sure, read the appropriate sections before making your selection.

☒ Life sciences ☐ Behavioural & social sciences ☐ Ecological, evolutionary & environmental sciences

For a reference copy of the document with all sections, see nature.com/documents/nr-reporting-summary-flat.pdf

Life sciences study design

All studies must disclose on these points even when the disclosure is negative.

Sample size	All samples were chosen based on previous experience and were a minimum of 3 independent experiments.
Data exclusions	For microscopy image analysis any field containing obvious artefacts were removed.
Replication	All experiments were repeated a minimum of three times frequently by two or more different operators. All attempts at replication were successful.
Randomization	No randomization was undertaken, however experiments were independently repeated and plate positions varied between researchers.
Blinding	No blinding was undertaken, however experiments were independently repeated and analyzed in unbiased manner by at least 2 researchers.

Reporting for specific materials, systems and methods

We require information from authors about some types of materials, experimental systems and methods used in many studies. Here, indicate whether each material, system or method listed is relevant to your study. If you are not sure if a list item applies to your research, read the appropriate section before selecting a response.

Materials & experimental systems

n/a	Involved in the study	
☐	☒	Antibodies
☐	☒	Eukaryotic cell lines
☒	☐	Palaeontology and archaeology
☒	☐	Animals and other organisms
☒	☐	Human research participants
☒	☐	Clinical data
☒	☐	Dual use research of concern

Methods

n/a	Involved in the study	
☒	☐	ChIP-seq
☒	☐	Flow cytometry
☒	☐	MRI-based neuroimaging

Antibodies

Antibodies used	anti-GST-HRP - GeneTex, GTX114099; anti-6X His tag (HRP) - Abcam, ab1187; anti-KRAS+HRAS+NRAS - Abcam, ab206969; anti-ERK - Abcam, ab184699; phospho-ERK - Abcam, ab76299; anti-pERK - Cell Signalling Technology 4370; Goat anti-Rabbit HRP- Cell Signalling Technology, CST7074S; Goat anti-Rabbit AlexaFluor 546 - Molecular Probes, A11010; Goat anti-Rabbit AlexaFluor 568 - Molecular Probes, A110101.
Validation	GTX114099: Immunoblotting, validated with purified GST protein - https://www.genetex.com/Product/Detail/GST-tag-antibody-HRP/GTX114099<br>ab1187: Immunoblotting, validated with purified His-tagged Affimers - https://www.abcam.com/hrp-6x-his-tag-antibody-ab1187.html<br>ab206969: Immunoblotting, validated with purified KRas protein - https://www.abcam.com/ras-antibody-epr18713-13-ab206969.html<br>ab184699 : Immunoblotting, Knock-out validated by manufacturer- https://www.abcam.com/erk1--erk2-antibody-epr17526-ab184699.html<br>ab76299: Immunoblotting, manufacturer-validated with use of lambda phosphatse- https://www.abcam.com/erk1-pt202py204--erk2-pt185py187-antibody-ep197y-ab76299.html<br>CST 4370: Immunofluorescence - https://www.cellsignal.co.uk/products/primary-antibodies/phospho-p44-42-mapk-erk1-2-thr202-tyr204-d13-14-4e-xp-rabbit-mab/4370?_=1621279740787&Ntt=4370&tahead=true

Eukaryotic cell lines

Policy information about cell lines

Cell line source(s)	HEK293, Panc 10.05 and NCI-H460 cells were purchased from ECACC, UK. RAS-expressing mouse embryonic fibroblasts

Cell line source(s)	(MEFs) were from William Burgen at Fredrick National Laboratory, Maryland, USA. SW620 cells were from Professor Mark Hull, University of Leeds, UK (originally purchased from ATCC).
Authentication	All cells were authenticated by STR profiling.
Mycoplasma contamination	All cell lines were mycoplasma negative as tested in house using the Lonza MycoAlert kit.
Commonly misidentified lines (See ICLAC register)	None
